# Guidelines for the Treatment of Abdominal Abscesses in Acute Diverticulitis: An Umbrella Review

**DOI:** 10.3390/jcm12175522

**Published:** 2023-08-25

**Authors:** Roberto Cirocchi, Francesca Duro, Stefano Avenia, Matteo Capitoli, Giovanni Domenico Tebala, Massimiliano Allegritti, Bruno Cirillo, Gioia Brachini, Paolo Sapienza, Gian Andrea Binda, Andrea Mingoli, Piergiorgio Fedeli, Riccardo Nascimbeni

**Affiliations:** 1 Department of Medicine and Surgery, S. Maria Hospital, University of Perugia, 05100 Terni, Italy; roberto.cirocchi@unipg.it (R.C.); durofrancesca3@gmail.com (F.D.); matteo.capitoli@studenti.unipg.it (M.C.); 2 Department of Digestive and Emergency Surgery, AOSP of Terni, 05100 Terni, Italy; g.tebala@aospterni.it; 3 Department of Radiology, AOSP of Terni, 05100 Terni, Italy; m.allegritti@aospterni.it; 4 Emergency Department, Policlinico Umberto I, Sapienza University, 00185 Rome, Italy; gioia.brachini@uniroma1.it (G.B.); andrea.mingoli@uniroma1.it (A.M.); 5 Department of Surgery, Sapienza University, Viale del Policlinico 155, 00161 Rome, Italy; paolo.sapienza@uniroma1.it; 6 General Surgery, Biomedical Institute, 16152 Genoa, Italy; gianbinda1@gmail.com; 7 School of Law, University of Camerino, 62032 Camerino, Italy; piergiorgio.fedeli@unicam.it; 8 Department of Molecular and Translational Medicine, University of Brescia, 25121 Brescia, Italy; riccardo.nascimbeni@unibs.it

**Keywords:** percutaneous drainage, diverticular abscess

## Abstract

Background: This systematic umbrella review aims to investigate and provide an analysis of guidelines regarding the treatment of diverticular abscesses. Material and methods: A systematic literature search was performed using the Cochrane Overviews of Reviews model and the ‘Clinical Practice Guidelines’; at the end of initial search, only 12 guidelines were included in this analysis. The quality of the guidelines was assessed by adopting the “Appraisal of Guidelines for Research and Evaluation II” (AGREE II). The comparative analysis of these guidelines has highlighted the presence of some differences regarding the recommendations on the treatment of diverticular abscesses. In particular, there are some controversies about the diameter of abscess to be used in order to decide between medical treatment and percutaneous drainage. Different guidelines propose different abscess diameter cutoffs, such as 3 cm, 4–5 cm, or 4 cm, for distinguishing between small and large abscesses. Conclusions: Currently, different scientific societies recommend that diverticular abscesses with diameters larger than 3 cm should be considered for percutaneous drainage whereas abscesses with diameters smaller than 3 cm could be appropriately treated by medical therapy with antibiotics; only a few guidelines suggest the use of percutaneous drainage for abscesses with a diameter greater than 4 cm. The differences among guidelines are the consequence of the different selection of scientific evidence. In conclusion, our evaluation has revealed the importance of seeking new scientific evidence with higher quality to either confirm, reinforce or potentially weaken the existing recommendations from different societies.

## 1. Introduction

Guidelines are very important in daily clinical practice and are indeed the basis of surgical protocols. Their use—or misuse—has consequences, and not only in healthcare. For instance, “clinical practice guidelines” (CPGs), have a dual role in malpractice litigation: defensive (exculpatory evidence) or accusatory (inculpatory evidence). In the absence of evidence (“balance of probabilities” or “preponderance of evidence”), the claim is based only in the different opinions of experts.

In recent years, several guidelines on the treatment of acute complicated diverticulitis of the colon have been published by different scientific societies. Sometimes, however, the recommendations, for the management of abdominal abscesses in particular, are conflicting. This may be due to the different literature evidence used in the guidelines. Moreover, most of this evidence is low-level.

In the treatment of diverticular disease, guidelines may lead to operational difficulties in diagnostic/therapeutic choices and/or medico-legal evaluations due to vagueness of scope, non-uniformity of guidelines for the same topic or lack of updating in relation to the scientific literature. In the past few decades, various guidelines have been proposed by scientific societies, and their role has increased, to translate the best available evidence into common clinical practice. Well-crafted recommendations have progressively improved diagnostic accuracy and the effectiveness of therapy through standardization. This cultural revolution has led to an improvement in the quality of health systems and a reduction in healthcare variations, but in some cases, the impact of conflicting evidence in clinical practice is associated with malpractice claims linked to diverticular disease treatment. 

The goal of this review is to provide an analysis of guidelines regarding the treatment of diverticular abscesses [1], summarizing the evidence from the best available practice.

## 2. Materials and Methods

We used the Cochrane Overviews of Reviews model [1] to search the ‘*Clinical Practice Guidelines*’ in PubMed, SCOPUS, Web of Science, National Institute for Health and Care Excellence (NICE), Scottish Intercollegiate Guidelines Network (SIGN), Guidelines International Network (GIN) and the Cochrane Library.

The search strategy was carried out using the following keywords: “guideline”, “best practice”, “recommendations”, “consensus”, “acute diverticulitis” and “acute colonic diverticulitis”, in various combinations with the Boolean operators AND, OR and AND NOT. The search for relevant studies was performed from January 2010 to April 2023. The checklist of PRISMA-ScR (Preferred Reporting Items for Systematic Reviews and Meta-Analyses extension for Scoping Reviews) was followed [2].

The PubMed function “related articles” was used to extend the search. We also performed a hand-search of bibliographies of included studies, to identify further potentially eligible investigations. 

Inclusion criteria were all types of guidelines about the management of abdominal abscess from acute colonic diverticulitis; the search strategy applied to PubMed is described in Appendix A. Language restriction was applied at only English language because the majority of relevant and high-quality studies related to the research question are available in English, which may lead to a significant reduction in language bias.

All studies were independently assessed for eligibility by two reviewers (RC and GT), and eventual controversies were resolved by a consensus among the reviewers. The reviewers are two emergency consultant surgeons dedicated and experienced in colorectal surgery.

The following data were independently extracted from included guidelines: first author name, scientific society promoted, country, year of publication, statement and consensus levels of agreement.

The quality of the guidelines was assessed by adopting the 2017 updated version of the “Appraisal of Guidelines for Research and Evaluation II” (AGREE II) [3]. 

A total of 23 items divided into 6 dimensions were analyzed for each guideline.

These 6 domains assess, for Domain 1, the general goals and scopes of application of the guideline; Domain 2 analyzes the study participants (stakeholders); Domain 3 assesses the methodological rigor with which the guideline was produced; Domain 4 assesses the clarity of exposition, that is, how easily identifiable and understandable are the recommendations presented; Domain 5 assesses applicability; the last one analyzes editorial independence.

Each item was rated on a scale from 1 (strongly disagree) to 7 points (strongly agree). The score of each dimension is equal to the score of the various items contained in each dimension, calculated as a percentage according to the formula:

$$\frac{\mathrm{Score}\;\mathrm{achieved}-\mathrm{lowest}\;\mathrm{possible}\;\mathrm{score}}{\mathrm{Highest}\;\mathrm{possible}\;\mathrm{score}-\mathrm{lowest}\;\mathrm{possible}\;\mathrm{score}}\times 100$$

Based on the results obtained, recommendation level A (strong recommendation) was assigned if all dimensions scored above 60%, or recommendation level B (weak recommendation), if at least one dimension scored below 60% [3].

## 3. Results

At the end of the initial search of titles and abstracts, 34 full texts were screened for relevance; only 12 guidelines (21 full text) were included in the review [4,5,6,7,8,9,10,11,12,13,14,15,16,17,18,19,20,21,22,23,24] (Figure S1).

The following data were independently extracted from included guidelines: first author name, scientific society promoted, country, year of publication, statement, consensus levels of agreement (Table 1a), direction of recommendations (Table 1b) and strength of recommendations (Table 1c).

The reasons of exclusion of ten guidelines were the following:

- the topic of guidelines is diverticular disease, but the authors do not report the treatment of abscess (three articles) [25,26,27];
- the guidelines were not written in the English language (five articles) [28,29,30,31,32];
- the authors performed an analysis only on elderly patients or patients underwent laparoscopic treatment (two articles) [33,34].

The evaluation of guideline quality was performed with AGREE II. [35]. 

According to the adopted criteria, seven guidelines reach a strong (Level A) recommendation (Kruis [5,6], Schultz [8], Sartelli [9], Hall [16], NICE [24], Francis [17] and Cuomo [21]) (SDC 1) whereas five guidelines reach a weak (Level B) recommendation (Qaseem [4], Pietrzak [19], Binda [20], Andeweg [22] and Andersen [23]) (SDC 2).

Focusing on the quality of each of the six dimensions according to the adopted score:

- Dimension 1—General objectives and areas of application (item 1–3): all guidelines were high quality, totaling a percentage higher than 60% (SDC 3).
- Dimension 2—Involvement of stakeholders (item 4–6): most guidelines, except those of Binda [20] with 33.3% and Andeweg [22] with 44.4%, totaled a score above 60% (SDC 4).
- Dimension 3—Methodological rigor (item 7–14): all guidelines but that of PieTrzak [19], with 41.6%, scored higher than 60% (SDC 5).
- Dimension 4—Expository clarity by language, structure and format (item 18–21): all except one guideline (Qaseem [4] with 45.8%) scored more than 60% (SDC 6).
- Dimension 5—the applicability by analyzing the possible barriers and factors facilitating the implementation of the guideline, the possible strategies to favor its adoption, the implication for the economic resources resulting from the application of the guideline (items 18–21): eight guidelines (Kruis [5,6], Schultz [8], Sartelli [9], Hall [16], NICE [24], Francis [17], Cuomo [21] and Andeweg [22]) reached a high level of quality, totaling scores above 60%, whereas the remainder (Qaseem [4], Pietrzak [19], Binda [20] and Andersen [23]) achieved a weak level of recommendation, scoring below 60% (SDC 7).
- Dimension 6—Editorial independence (Items 22–23): most guidelines, except those of Pietrzak [19], Binda [20], Andeweg [22] and Andersen [23], achieved a good level of recommendation, scoring above 60% (SDC 8).

In the table we find the extended results of the evaluation (SDC 19).

### Analysis of Conflicting Recommendations

The comparative analysis of these guidelines has brought to light several differences concerning the recommendations for treating diverticular abscesses. Specifically, there are controversies surrounding the appropriate abscess diameter that should be considered when deciding between medical treatment and percutaneous drainage (Table 2).

A well-defined cut-off for performing percutaneous drainage was not established in seven guidelines. Four of these guidelines are earlier versions of the ASCRS [12,13,14,15], and one is a previous version of the EAES [18].

This diameter was standardized in four groups, two of which were overlapping:

- Less than 3 cm;
- between 3–4 cm;
- less than 4 cm;
- between 4–5 cm.

Currently, most scientific societies (DGVS [5] and DGAV [6], ASCRS [16], ESCP [8], NICE [24], PSG and TChP [19]) recommend that diverticular abscesses with diameters larger than 3 cm should be considered for percutaneous drainage whereas abscesses with diameters smaller than 3 cm could be appropriately treated by medical therapy with antibiotics, only.

In contrast, other guidelines, suggest the use of percutaneous drainage for abscesses with a diameter greater than 4 cm (EAES and SAGES 2018) [17] and 5 cm (SICCR 2015) [20]. The differences among guidelines are the consequence of the different selection of scientific evidence. 

The ESCP 2020 recommendations, based on a systematic review and a multicenter study, suggest performing percutaneous drainage in abscesses larger than 3 cm in diameter [36,37].

- ESCP 2020 [8]: “Although the role of percutaneous drainage of abscesses in acute diverticulitis is not completely clear, it may be considered in patients with an abscess larger than 3 cm. Emergency surgery should be kept as last resort for patients failing other nonsurgical treatments”.

In addition, the LG DGVS/DGAV 2022 supports this recommendation.

- DGVS and DGAV 2022 [6,7]: “To distinguish between micro and macro abscesses, a threshold value of approximately 3 cm can be applied, since this reflects the possibility of interventional drainage and the risk of recurrence correlates with the size of the abscess”. “Larger retroperitoneal or paracolic abscesses (>3 cm) can be interventionally drained (sonography, CT)”.

This opinion is also the one reported in the ASCRS 2020 Guidelines, which are based on two prospective studies [38,39].

- ASCRS 2020 [16]: “Image-guided percutaneous drainage is usually recommended for stable patients with abscesses >3 cm in size”. “Evidence level moderate-quality evidence 1B, recommendation grade: strong recommendation”.

Finally, we should highlight that even the NICE guidelines, based on 5 studies [40,41,42,43,44], suggest performing percutaneous drainage in abscesses with a diameter > 3 cm.

- NICE 2019 [24]: “The committee agreed that if percutaneous drainage is an anatomically feasible option this could be considered alongside a discussion with the patient about the risks and benefits of surgery. In people with a CT-confirmed diverticular abscess, re-imaging may be considered if the condition does not improve clinically or if there is deterioration”.

In Suppl 6 of the consensus guideline of the EAES and SAGES 2018 [17] states that “Twenty-five papers covering 21,656 patients reported on the non-resectional management of diverticular abscesses (Hinchey Ib-II) and/or perforated diverticulitis” and also suggests percutaneous drainage in abscess with a diameter greater than 4 cm.

In identifying the diameter of the abscess, these guidelines are based largely on systematic reviews by Gregersen and on another studies [36,41,45]

In conclusion, only WSES 2020, based on 5 studies, reported a major cutoff of 4–5 cm as the lower abscess diameter for percutaneous drainage.

- WSES 2020 [9]: “For patients with a small (<4–5 cm) diverticular abscess, we suggest an initial trial of non-operative treatment with antibiotics alone (weak recommendation based on low- quality evidence, 2C). We suggest to treat patients with large abscesses with percutaneous drainage combined with antibiotic treatment; whenever percutaneous drainage of the abscess is not feasible or not available, we suggest to initially treat patients with large abscesses with antibiotic therapy alone, clinical conditions permitting. Alternatively, an operative intervention is required”.

## 4. Discussion

The current management of diverticular abscesses requires a multidisciplinary approach involving infectious disease specialists, interventional radiologists and surgeons. The therapeutic decisions are based on CT scan results, which provide accurate information about the size and location of the abscesses and help determine the appropriate treatment approach.

For small sized diverticular abscesses, intravenous antibiotic therapy is recommended. On the other hand, larger abscesses may require percutaneous drainage if anatomically accessible. 

Surgery is reserved for cases where percutaneous drainage fails or for large abscesses that cannot be accessed through percutaneous drainage and are unresponsive to antibiotic therapy. 

However, there is controversy among different guidelines regarding the cutoff diameter for abscesses that require percutaneous drainage. In fact, the definition of “small” and “large” abscesses varies among the guidelines, leading to inconsistent recommendations. 

For example, one of the most recent guidelines, from the German Societies for Gastroenterology and Visceral Surgery (DGVS/DGAV), identifies a diameter of 3 cm as the value for distinguishing between micro and macro abscesses. This cutoff is also shared by other societies, such as the ASCRS (American Society of Colon and Rectal Surgeons) and NICE (National Institute for Health and Care Excellence). However, differing from these is the World Society of Emergency Surgery (WSES), which reported a cutoff of 4–5 cm.

Some studies, including one by Lambrichts, have shown worse outcomes for patients undergoing percutaneous drainage compared to those who received medical therapy alone; these results may be influenced by selection bias, as patients in worse general conditions are more likely to be enrolled in percutaneous drainage [37,38].

Another study conducted by Elagili et al. sought to evaluate the difference between treatment with antibiotic therapy alone and percutaneous drainage in abscesses of diameter larger than 3 cm. This showed that selected patients could be treated initially with antibiotics alone without any negative consequences on their outcomes [39].

However, a retrospective analysis conducted by Buchwald et al. on patients admitted to Christchurch Hospital evaluated the long-term outcomes of conservative treatment in diverticular abscesses (Hinchey I and II). It was found that the number of recurrences was higher in patients who were treated with initial conservative treatment (antibiotics ± percutaneous drainage) than in those who underwent surgery [44].

The NICE guidelines suggest considering percutaneous drainage only for abscesses larger than 3 cm due to technical difficulties in performing the procedure on smaller abscesses [24].

However, these guidelines acknowledge that the evidence supporting this recommendation is of very low quality due to selection bias and other limitations. Meta-analyses reported by NICE have evaluated outcomes without stratifying patients based on different abscess diameters [41,44]. Therefore, there is a need for new large-scale studies to determine the appropriate diameter cutoff for percutaneous drainage.

When analyzing the recommendations in the included guidelines together, there is a certain degree of overlap between studies on which the various groups have based their recommendations regarding the treatment of diverticular abscesses. The systematic review by Gregersen et al. [36], was considered in two LGs (NICE 2019 and EAES/SAGES 2018) and the research of Siewert et al. [42] was considered in the WSES 2020 and NICE 2019 guidelines.

## 5. Conclusions

The treatment of abdominal abscesses in patients with acute colonic diverticulitis is a significant therapeutic challenge. The approach has shifted from surgical intervention to non-surgical, minimally invasive management over the years. This change is attributed to advancements in technology, improved intensive care monitoring, the use of computed axial tomography, and the introduction of minimally invasive techniques such as percutaneous US/CT-guided drainage.

Various national and international societies have attempted to provide treatment recommendations, but there are conflicting opinions, especially regarding the abscess cutoff diameter for intervention. Guidelines often have a low degree of recommendation due to the low quality of evidence, primarily stemming from selection bias and imprecision.

Different guidelines propose different abscess diameter cutoffs, such as 3 cm, 4–5 cm, or 4 cm, for distinguishing between small and large abscesses. In the end, the diameter that is still the source of debate is 3 cm, while everyone agrees on performing percutaneous drainage, where practicable, for larger abscesses with a diameter from 4 to 5 cm.

In conclusion, our evaluation has revealed the importance of seeking new scientific evidence with higher quality to either confirm, reinforce or potentially weaken the existing recommendations from different societies.

This pursuit will undoubtedly result in more accurate and shared recommendations, which can resolve the controversies in various guidelines. Most importantly, it can lead to improved treatment outcomes for patients with abdominal abscess from acute colonic diverticulitis.

## Figures and Tables

**Table 1:** Characteristics of Guidelines included.

**(a) Guidelines Included with Consensus Levels of Agreement.**				
** Guideline **		** Data of Publication **	** Previous Editions **	** Levels of Agreement **
The National Institute for Health and Care Excellence	NICE	2019 (NICE [24])		Standards committee
Gastrointestinal and Endoscopic Surgeons	SAGES			Consensus meeting
European Association for Endoscopic Surgery	EAES	2018 (Francis [17])	2009 (Köhler [18])	Consensus meeting
Polish Society of Gastroenterology and the Association of Polish Surgeons	PSG TChP	2015 (Pietrzak [19])		Expert panel
Italian Society of Colon and Rectal Surgery	SICCR	2015 (Binda [20])		Expert panel
Italian Study Group of Diverticular Disease	GRIMAD	2014 (Cuomo [21])		Consensus meeting
Netherlands Society of Surgery	NTVG	2013 (Andeweg [22])		Expert panel
Danish Surgical Society	DKS	2012 (Andersen [23])		Expert panel
**(b) Guidelines Included with Direction of Recommendation.**				
**Guideline**		**Data of Publication**	**Previous Editions**	**Direction of Recommendation**
American College of Physicians	ACP	2022 (Qaseem [4])		Favors percutaneous drainage in abscesses > 4 cm
German Societies for Gastroenterology and Visceral Surgery	DGVS DGAV	2022 (Kruis [5,6])	2014 (Kruis [7])	Favors percutaneous drainage in abscesses > 3 cm
European Society of Coloproctology	ESCP	2020 (Schultz [8])		Favors percutaneous drainage in abscesses > 3 cm
World Society of Emergency Surgery	WSES	2020 (Sartelli [9])	2016 (Sartelli [10]), 2011 (Sartelli [11])	Favors percutaneous drainage in abscesses > 4–5 cm
American Society of Colon and Rectal Surgeons	ASCRS	2020 (Hall [16])	2014 (Feingold [15]), 2006 (Rafferty [14]), 2000 (Wong [13]), 1995 (Roberts [12])	Favors percutaneous drainage in abscesses > 3 cm
The National Institute for Health and Care Excellence	NICE	2019 (NICE [24])		Consider percutaneous drainage alongside a discussion with the patient about the risks and benefits of surgery.
Gastrointestinal and Endoscopic Surgeons	SAGES	2018 (Francis [17])	2009 (Köhler [18])	Favors percutaneous drainage in abscesses > 4 cm, those that do not resolve on antibiotics
European Association for Endoscopic Surgery	EAES	2018 (Francis [17])	2009 (Köhler [18])	
Polish Society of Gastroenterology and the Association of Polish Surgeons	PSG	2015 (Pietrzak [19])		Favors percutaneous drainage in abscesses > 3 cm
Italian Society of Colon and Rectal Surgery	SICCR	2015 (Binda [20])		Favors percutaneous drainage in abscesses > 4 cm
Italian Study Group of Diverticular Disease	GRIMAD	2014 (Cuomo [21])		Favors percutaneous drainage in abscesses > 4 cm
Netherlands Society of Surgery	NTVG	2013 (Andeweg [22])		Favors percutaneous drainage combined with antibiotics in abscesses > 4–5 cm
Danish Surgical Society	DKS	2012 (Andersen [23])		Favors percutaneous drainage in abscesses > 3 cm
**(c) Guidelines Included with Strength of Recommendations.**				
** Guideline **			** Strength of Recommendation **	** Level of Evidence **
American College of Physicians		ACP	Conditional recommendation	Evidence level low-certainty
German Societies for Gastroenterology and Visceral Surgery		DGVS DGAV	Recommendation grade 0	Evidence level 3
European Society of Coloproctology		ESCP	Conditional recommendation	Evidence level 3
World Society of Emergency Surgery		WSES	Weak recommendation	Evidence level 2C
American Society of Colon and Rectal Surgeons		ASCRS	Strong recommendation.	Evidence level 1B
The National Institute for Health and Care Excellence		NICE		Evidence level low
Gastrointestinal and Endoscopic Surgeons		SAGES	Recommendation weak.	Evidence level low
European Association for Endoscopic Surgery		EAES		
Polish Society of Gastroenterology and the Association of Polish Surgeons		PSG TChP		
Italian Society of Colon and Rectal Surgery		SICCR		Evidence level 2A
Italian Study Group of Diverticular Disease		GRIMAD	Recommendation C	Evidence level 3B
Netherlands Society of Surgery		NTVG		Evidence level 3
Danish Surgical Society		DKS	Recommendation C.	Evidence level III

**Table 2:** Diameter measurement of abscess in patients undergoing percutaneous drainage.

Diameter Not Reported	Diverticular Abdominal Abscess Diameter > 3 cm	Diverticular Abdominal Abscess Diameter > 3–4 cm	Diverticular Abdominal Abscess Diameter > 4 cm	Diverticular Abdominal Abscess Diameter > 4–5 cm
DKS 2012 (Andersen [23]) EAES 2009 (Köhler [18]) ASCRS 2006 (Rafferty [14]) ASCRS 2000 (Wong [13]) ASCRS 1995 (Roberts [12])	DGVS and DGAV 2022 (Kruis [5,6]) ASCRS 2020 (Hall [16]) ESCP 2020 (Schultz [8]) NICE 2019 (NICE [24]) PSG and TChP 2015 (Pietrzak [19])	ASCRS 2014 (Feingold [15])	EAES and SAGES 2018 (Francis [17]) SICCR 2015 (Binda [20]) GRIMAD 2014 (Cuomo [21]) WSES 2011 (Sartelli [11]) DGVS and DGAV 2014 (Kruis [7])	WSES 2020 (Sartelli [9]) WSES 2016 (Sartelli [10]) NTVG 2013 (Andeweg [22])

## Data Availability

Not applicable.

## Supplementary Materials

The following supporting information can be downloaded at: https://www.mdpi.com/article/10.3390/jcm12175522/s1, Figure S1: Prisma flow chart of literature search.

## Appendix A

- #1. Diverticular Disease
- #2. Diverticular perforation
- #3. Peritonitis
- #4. Free Air
- #5. Abscess
- #6 #1 OR #2 OR #3 OR #4 OR #5
- #7. Guidelines
- #8. Consensus conference
- #9. Statement
- #10 #7 OR #8 OR #9
