# Supplementary figures and images for: Guidelines for the Treatment of Abdominal Abscesses in Acute Diverticulitis: An Umbrella Review
Source: J Clin Med. 2023 Aug 25;12(17):5522. doi: 10.3390/jcm12175522 (PMC10488020; doi:10.3390/jcm12175522)

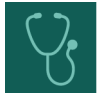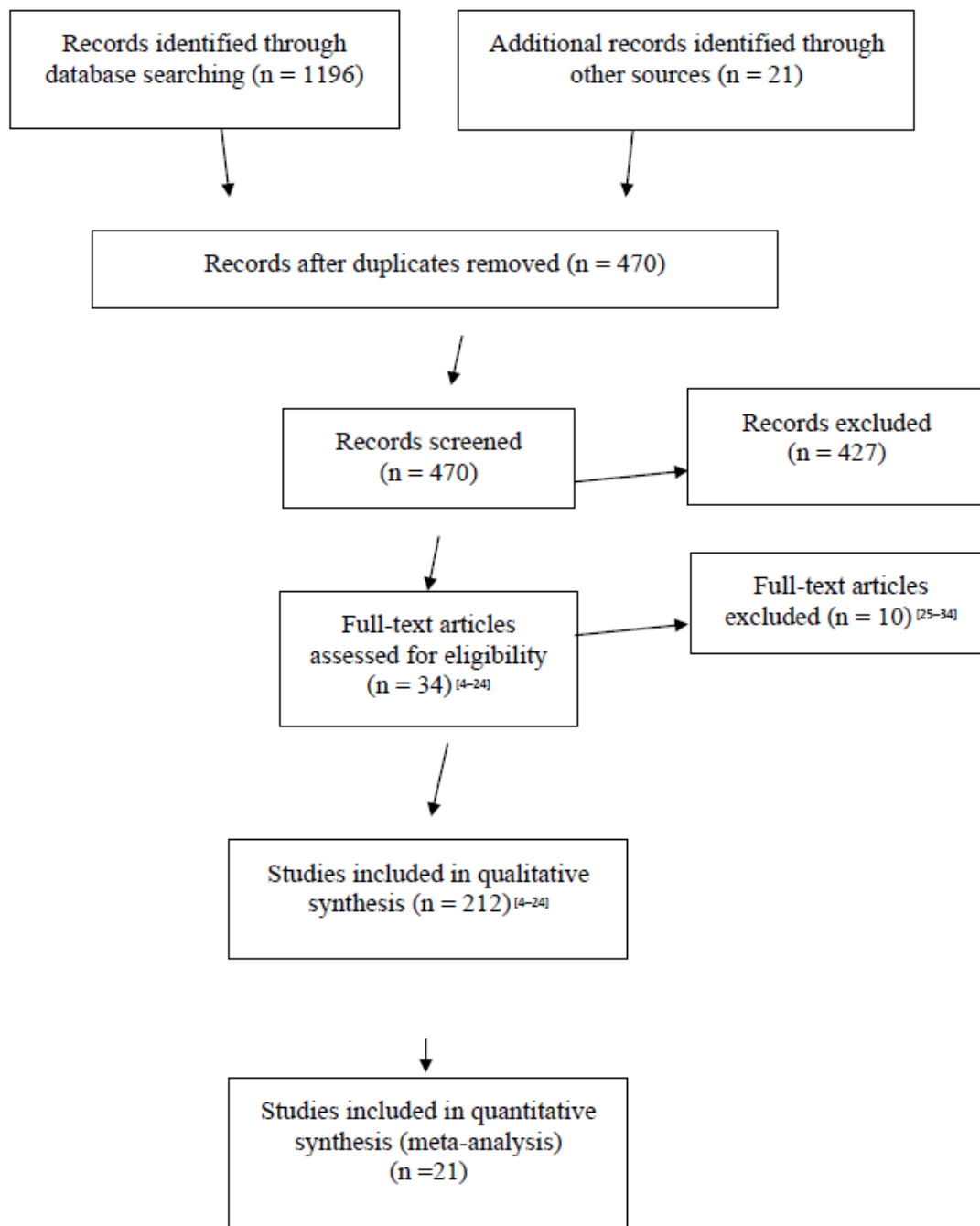

**Figure S1.** Prisma flow chart of literature search.

Supplement: Supplementary file 1 [file jcm-12-05522-s001.zip › jcm-2510736-supplementary.pdf]
